# Self-medication behavior among medical students: the roles of stress, self-efficacy, and health literacy

**DOI:** 10.1186/s12909-026-09240-5

**Published:** 2026-04-23

**Authors:** Abdul Basit, Marriyum Rashid Khan, Ayesha Fiaz Ud Din, Muskan Waheed, Marwa Hamid, Mahnoor Rehman, Syeda Khadijah Mahrukh, Muhammad Waqar Shahid, Fatima Sajjad, Kamil Ahmad Kamil

**Affiliations:** 1 Department of Medicine, Nowshehra Medical College, Nowshehra, Pakistan; 2 Department of Medicine, Khyber Medical University, Peshawar, Pakistan; 3 Department of Medicine, Khyber Medical College, Peshawar, Pakistan; 4 Internal Medicine Department, Mirwais Regional Hospital, Kandahar, Afghanistan

**Keywords:** Self-medication, Medical students, Stress, Self-efficacy, Health literacy, Pakistan

## Abstract

**Background:**

Self-medication, the use of medications without professional consultation, is prevalent among medical students and poses risks such as drug misuse and delayed diagnosis. This study examined the roles of stress, self-efficacy, and health literacy in self-medication behaviours among undergraduate medical students in Pakistan.

**Methods:**

A cross-sectional study was conducted from April to May 2025 at Nowshehra Medical College, involving 241 students (158 females and 83 males) selected through a convenience sampling method. A self-modified structured questionnaire was used to assess health literacy (nine items), self-efficacy (six items), stress (ten items), and self-medication behaviour. Data were analyzed using SPSS v30.0, employing t-tests to compare sex differences and binary logistic regression to identify predictors of the outcome. Ethical approval was obtained (Ref. 30/ERB/NMC).

**Results:**

No sex differences were found in health literacy (*p* = 0.630) or self-efficacy (*p* = 0.156), but females reported higher stress (*p* < 0.001, Cohen’s d = -0.59). Logistic regression models indicated that health literacy factors were not significant predictors (*p* > 0.05). However, significant predictors included reliance on past experiences (OR = 1.823, *p* = 0.003) and health-related stress (OR = 1.607, *p* = 0.007), while safe self-medication practices were protective (OR = 0.679, *p* = 0.027). The overall model explained 18.5% of the variance (Nagelkerke R² = 0.185) but had low classification accuracy (47.9–49.2%). Visualisations, including bar charts, ROC curves, box plots, and heatmaps, highlighted the stress disparities and predictor strengths.

**Conclusion:**

Unsupervised medication use and stress drive self-medication among medical students. Targeted interventions should promote safe practices, address gender-specific stressors, and enhance health literacy to mitigate risks.

**Supplementary Information:**

The online version contains supplementary material available at 10.1186/s12909-026-09240-5.

## Introduction

Self-medication, defined as the intake of drugs without professional medical advice, is a widespread practice among young adults and university students. Although convenient, this practice is associated with serious risks, such as self-diagnosis errors, drug misuse, and physical injury [1]. Self-medication is highly frequent among medical students, who are exposed to medical knowledge early, as they tend to assess health-related issues as part of their domain [2]. However, psychosocial and health literacy aspects may play a role in the decision to self-medicate, possibly increasing the risks. The current study aimed to explore the relationship between stress, self-efficacy, and health literacy and the practice of self-medication among undergraduate students at Nowshehra Medical College, Pakistan, using a cross-sectional survey.

Self-medication is becoming an increasing public health problem, and its prevalence varies significantly among different populations worldwide. Research has shown that self-medication among university students ranges from 38% to 92% globally, depending on the country studied, with higher rates in low- to middle-income countries with restricted access to healthcare [3–5]. This is also supported by the ready availability of over-the-counter (OTC) drugs and an acceptable tradition of self-care in the form of self-medication in Pakistan, where it is approximately 76% in medical students [4]. This practice involves the deviation of established clinical guidelines and evidence-based practices [5, 6] and is primarily influenced by convenience, time pressure, and self-assessed knowledge about medications [2, 7, 8] which result in adverse outcomes. Improper medication use can cause drug resistance, toxicity, and delayed detection of underlying diseases, highlighting the importance of identifying the factors affecting this behaviour [5, 9].

Stress-related negative health behaviours in health science schools are more pronounced than in other disciplines, and in some cases, stimulants may even be abused. The high standard of learning in medical school and demanding academic requirements presumably prompt students to resort to self-medication as a coping technique [10]. Studies have shown that stress is related to the more frequent use of analgesics, sedatives, and other drugs without prescriptions, as subjective improvements in symptomatology are easily obtained this way [11]. A study among medical students in India revealed that 62% of them consumed unprescribed drugs to alleviate stress-related symptoms, such as body pain and insomnia [7]. This implies that stress could contribute not only to self-medication but also to the worsening of high-risk health behaviours, which invites further research in specific subgroups.

Self-efficacy, which is an individual’s belief about their capability, is the other most crucial predictor of health behaviour [12]. Regarding the practice of self-medication, those with high self-efficacy may have the self-confidence to choose and use medication without professional advice. This may contribute to the risk of self-medication among them. According to Bandura’s self-efficacy theory, individuals who have confidence in their ability to manage their health are more inclined to engage in proactive health management; however, they may also become overconfident when making medical treatment decisions [13]. Some studies have demonstrated that medical students generally possess high self-efficacy in health-related activities as a result of their education, which may lead to self-medication [8]. For example, in a study conducted in Ethiopia, students with a high level of self-efficacy were 34% more likely to engage in self-medication than those with a low level of self-efficacy [14]. However, the correlation between self-efficacy and safe self-medication practices has not yet been explored properly, especially in Pakistan.

Health literacy (HL), which refers to the capacity to access, understand, and use information to make informed health decisions, is a critical determinant of health behaviours. Low health literacy is associated with medication non-adherence and misuse, whereas sufficient health literacy enables patients to make informed decisions [15]. Health literacy is generally assumed to be high among medical students, given their academic exposure; however, this assumption does not necessarily reflect safe behaviours in practice. In Saudi Arabia, self-medication by medical students with high health literacy was very high (68%), and they often misunderstood drug instructions [16]. This paradox may imply that health literacy has a limited influence on self-medication and that affect and/or overconfidence would offset enlightenment or informed choice [15, 17]. In Pakistan, significant differences in literacy levels have been observed, and identifying this as a factor in self-medication habits among medical students is essential for targeted interventions.

The interaction between stress, self-efficacy, and health literacy in self-medication is dynamic and situation-dependent. Although urgent suffering due to illness may lead to stress and a desire for immediate symptom relief, self-efficacy and health literacy play a role in both deciding and implementing self-medication [13, 15, 17]. For example, students with high stress and low health literacy might be more likely to use medications inappropriately [15, 18]. In contrast, those with high self-efficacy are likely to self-medicate with confidence but not always with accuracy. While studies have examined these factors individually, few have investigated them together within a single model, specifically among medical students in Pakistan [4, 7, 14]. This void in the literature underscores the need for a thorough examination in the form of evidence-based educational and policy initiatives. This study is guided by the Health Belief Model and Bandura’s Self-Efficacy Theory. The HBM suggests that perceived stress may increase the likelihood of self-medication due to a greater perceived need for symptom relief. Bandura’s theory proposes that individuals with higher self-efficacy may be more likely to independently manage their health, which may increase self-medication behaviour. In contrast, health literacy is expected to reduce self-medication by enabling informed and safe medication decisions.

- H1: Higher stress levels are related to increased self-medication behaviour among undergraduate students.
- H2: Higher self-efficacy is related to increased self-medication behaviour due to better confidence in self-management.
- H3: Higher health literacy is related to safer and reduced self-medication practices.

Pakistan provides a unique context for examining self-medication, given its health system problems, such as low utilisation of primary healthcare services, easy over-the-counter availability of drugs, and cultural preferences supporting self-care [4]. As future healthcare providers, medical students represent a significant target population for investigation, given that their behaviours will have implications for future patient care and counselling. The high level of self-medication in this population raises questions about their knowledge of safe drug use and habituation to risky health practices [2]. Furthermore, stressful and challenging learning environments in medical schools are likely to reinforce these practices, and the underlying psychological and cognitive factors need to be examined [10].

This study aims to fill these gaps by exploring the roles of stress, self-efficacy, and health literacy in self-medication behaviour among undergraduate students at Nowshehra Medical College. Using a cross-sectional study design and a pre-tested questionnaire, this study aims to estimate the associations of these variables and the relevant predictors of self-medication. We anticipate that our results will elucidate the causes of self-medication and guide interventions that may promote health and safety among medical students. More specifically, this study will explore whether stress exacerbates self-medication and whether people with high self-efficacy will overconfidently self-medicate, as well as whether health literacy will temper or complement these relationships. Ultimately, this research aims to aid in the design of educational interventions that promote health literacy, stress management, and positive self-efficacy among young health professionals.

## Methods

### Study design

This cross-sectional study evaluated the influence of stress, self-efficacy, and health literacy on self-medication behaviour among undergraduate students at Nowshehra Medical College, Pakistan.

### Study setting

The study was carried out in Nowshehra Medical College, Pakistan, following approval from the Ethical Review Board (ERB) of Nowshehra Medical College (Reference No. 30/ERB/NMC). All methods were carried out in accordance with the Declaration of Helsinki. All participants provided informed consent, and confidentiality was ensured with the option of withdrawal at any time. The study was conducted over one month, from April to May 2025.

### Study population and sampling

The study population comprised 640 undergraduate medical students enrolled at Nowshehra Medical College. A non-probability convenience sampling method was employed to select 241 participants (158 females and 83 males), aged 18–30 years, from various academic years.

### Sample size calculation

The sample size was calculated using OpenEpi software, assuming an anticipated frequency of 50%, a margin of error of 8.83%, and a 95% confidence level [19].

### Inclusion and exclusion criteria

The inclusion criteria included undergraduate students of Nowshera Medical College (NMC) only and those who were willing to participate, while those who were not affiliated with NMC, and did not consent or had any illnesses that affected their self-medication behaviours were excluded.

### Data collection tool

Data were collected using a self-modified, structured, closed-ended questionnaire designed to assess three independent variables—knowledge of self-medication, stress-handling abilities, and health literacy—and one dependent variable, self-medication behaviour. The questionnaire was developed based on the study objectives and validated through a pilot study for which a sample size of 30 participants was collected at Nowshehra Medical College. These participants were excluded from the final analysis of (*n* = 241). The instrument demonstrated good internal consistency (Cronbach’s α = 0.88) to ensure its clarity and reliability. The instrument comprised 25 direct questions: nine for health literacy, ten for stress, and six for self-efficacy, all of which were scored on a Likert scale. The questions focused on general medication practices like over-the-counter (OTC) medications such as analgesics, antipyretics, and cough syrups, without referencing specific prescription drugs, to ensure broader applicability and to avoid inappropriate or unsafe medication use [20]. The questionnaire was administered electronically via Google Forms, and clear instructions were provided to the participants to ensure accurate responses. The full 25 items questionnaire is provided in Supplementary File 1.

Data was collected in a controlled manner. Although the questionnaire was distributed electronically via Google Forms using official student communication channels, including WhatsApp groups and other social media platforms, the elements of control were maintained by restricting the responses to one per participant, with participants completing the survey anonymously to minimise bias and the data collector was present in person to provide guidance and ensure completion. Also the study was conducted during a period of standard academic activity, excluding peak examination weeks. An Informed consent was obtained electronically before accessing the survey. The research team supervised the process to ensure compliance with the ethical standards.

### Statistical analysis

Data was exported from Google Forms to SPSS version 30.0 for further analysis. Descriptive statistics, including the means, medians, standard deviations, frequencies, and percentages, were calculated for both numerical and categorical variables. Independent samples t-tests were used to compare health literacy, self-efficacy, and stress scores by gender, with Levene’s test applied to assess the equality of variances. Binary logistic regression was employed to identify predictors of self-medication behaviour, with variables stratified by age, gender, and independent factors to explore their effects. Multicollinearity among the predictors was assessed to ensure model validity [21]. All statistical tests were two-tailed, with a significance level of *p* < 0.05.

### Ethical consideration

Ethical considerations were prioritised throughout this study. The ERB approval ensured adherence to ethical guidelines, and the participants’ right to privacy and voluntary participation were upheld. The data were stored securely and were accessible only to the research team. This methodology employed a quantitative cross-sectional design to evaluate the interplay between stress, self-efficacy, health literacy, and self-medication behaviour, thereby contributing to the understanding of health practices among students at Nowshera Medical College [22].

## Results

This cross-sectional study examined the roles of health literacy, self-efficacy, and stress in self-medication behaviour among 241 undergraduate medical students (158 females and 83 males) at Nowshera Medical College. Data were analyzed using descriptive statistics, independent samples t-tests, and binary logistic regression in SPSS v30.0. The results are summarised in Tables 1 and 2.

**Table 1:** Gender differences in health literacy, self-efficacy, and stress (T-test results)

Variable	Gender	N	Mean	SD	t	df	*p*-value	Mean Difference	95% CI	Cohen’s d
Health Literacy	Male	83	0.46	0.59	0.48	144.10	0.630	0.036	-0.11, 0.18	0.07
	Female	158	0.43	0.46						
Self-Efficacy	Male	83	0.39	0.54	-1.43	151.45	0.156	-0.10	-0.23, 0.04	-0.19
	Female	158	0.49	0.45						
Stress	Male	83	-0.02	0.75	-4.41	238	<0.001***	-0.41	-0.60, -0.22	-0.59
	Female	158	0.39	0.66						

Stress scores are reported as standardized Z-scores

^***^p < 0.001

**Table 2:** Logistic regression predictors of self-medication

**Variable**	**B**	**SE**	**Wald**	**df**	***p***-value	**OR**	**95% CI**
Health Literacy							
Read_instructions	0.044	0.152	0.084	1	0.772	1.045	0.775–1.409
Self-Efficacy							
Selfmedication_safe	-0.388	0.175	4.898	1	0.027*	0.679	0.482–0.957
Rely_experiences	0.600	0.201	8.906	1	0.003**	1.823	1.229–2.704
Stress							
Stress_concentration	-0.603	0.210	8.200	1	0.004**	0.547	0.362–0.827
Stress_health	0.474	0.174	7.386	1	0.007**	1.607	1.141–2.262
Highstress_selfmedication	0.331	0.153	4.693	1	0.030*	1.392	1.032–1.878

^*^p< 0.05, ^**^p< 0.01

### Health literacy

An independent sample t-test was used to assess sex differences in health literacy scores (Table 1). No significant difference was found between males (*n* = 83, M = 0.46, SD = 0.59) and females (*n* = 158, M = 0.43, SD = 0.46), t(144.10) = 0.48, *p* = 0.630 (two-tailed), with unequal variances assumed (F = 4.02, *p* = 0.046). The mean difference was 0.036 (95% CI: -0.11, 0.18), with a negligible effect size (Cohen’s d = 0.07, 95% CI: -0.19, 0.33), indicating that health literacy did not vary by gender.

Binary logistic regression was used to evaluate the predictors of health literacy for self-medication (Table 2). The model was significant (χ ² (5) = 35.932, *p* < 0.001), explaining 19.1% of the variance (Nagelkerke R² = 0.191). “read_instructions” did not significantly predict self-medication (*p* = 0.772, OR = 1.045, 95% CI: 0.775–1.409). The model demonstrated a good fit (Hosmer-Lemeshow χ² = 6.403, *p* = 0.602) and achieved a classification accuracy of 68.9% (84.1% for self-medication and 40.5% for non-self-medication), highlighting the critical role of unsupervised medication use.

### Self-efficacy

A t-test was used to examine the gender differences in self-efficacy scores (Table 1). No significant difference was observed between males (*n* = 83, M = 0.39, SD = 0.54) and females (*n* = 158, M = 0.49, SD = 0.45), t(151.45) = -1.43, *p* = 0.156 (two-tailed), with unequal variances (F = 4.25, *p* = 0.040). The mean difference was − 0.10 (95% CI: -0.23, 0.04), with a small-to-moderate effect size (Cohen’s d = -0.19, 95% CI: -0.47, 0.06).

Logistic regression analysis identified self-efficacy as a predictor of self-medication (Table 2). The model was significant (χ ² (7) = 50.716, *p* < 0.001), explaining 26.2% of the variance (Nagelkerke R² = 0.262). Significant predictors included “selfmedication_safe” (*p* = 0.027, OR = 0.679, 95% CI: 0.482–0.957), “rely_experiences” (*p* = 0.003, OR = 1.823, 95% CI: 1.229–2.704).The classification accuracy was 73.0% (89.2% for self-medication and 42.9% for non-self-medication), underscoring the influence of personal experience and unsupervised medication use.

### Stress

**Fig. 1:**
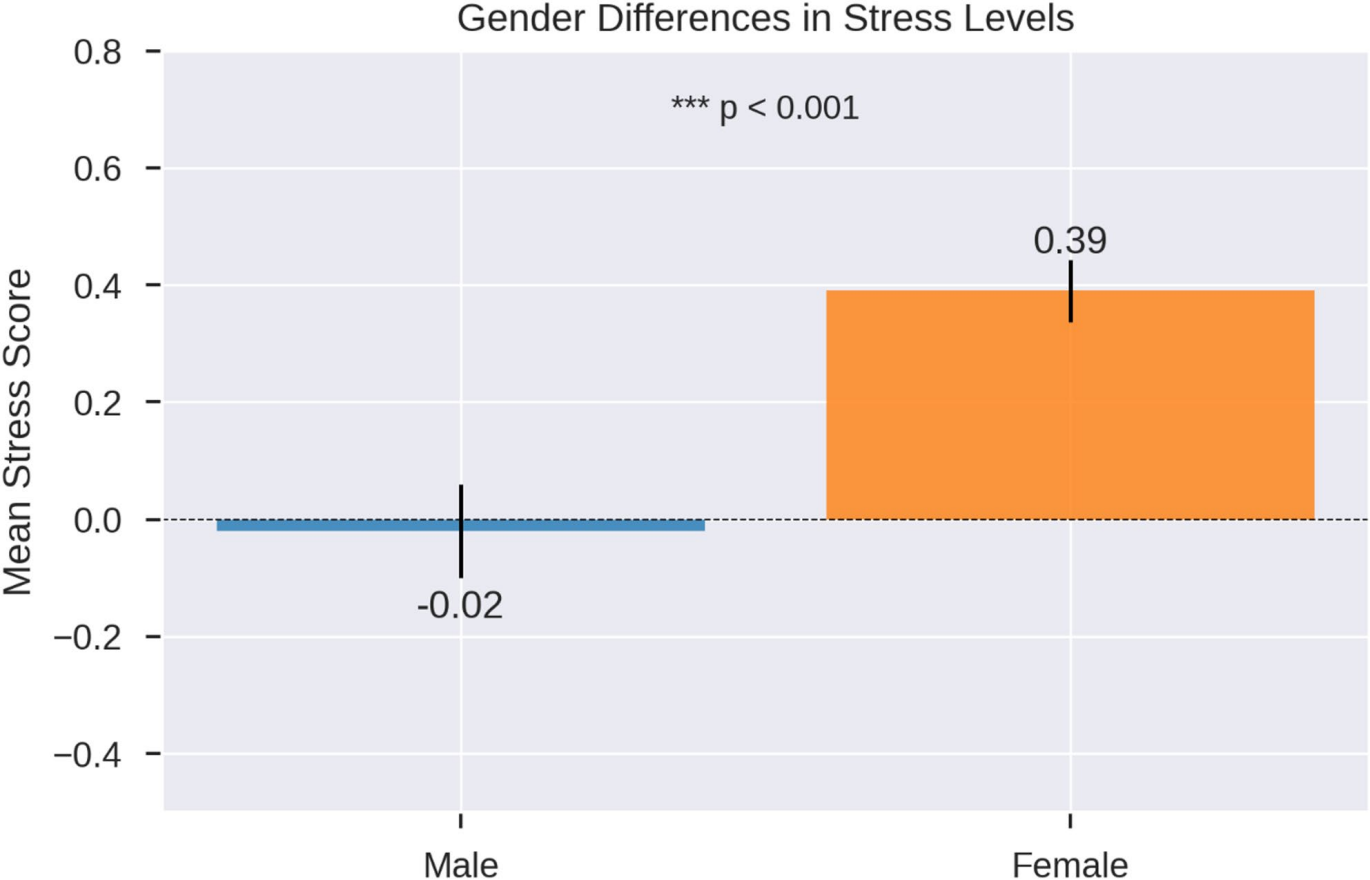
Gender differences in stress levels among undergraduate medical students at Nowshera Medical College. The chart displays the mean stress scores for males (M = -0.02, SE = 0.08, *n* = 83) and females (M = 0.39, SE = 0.05, *n* = 158), with error bars representing standard errors. A significant difference was observed (t(238) = -4.41, *p* < 0.001), with female students reporting higher stress levels than male students. This visualisation highlights the pronounced gender disparity in stress, which is a key factor influencing self-medication behaviour in this population

Gender differences in stress levels were evaluated using a t-test (Table 1, Fig. 1). Females (*n* = 158, M = 0.39, SD = 0.66) reported significantly higher stress than males (*n* = 83, M = -0.02, SD = 0.75), t(238) = -4.41, *p* < 0.001 (two-tailed), with equal variances assumed (F = 3.546, *p* = 0.061). The mean difference was − 0.41 (95% CI: -0.60, -0.22), with a moderate-to-large effect size (Cohen’s d = -0.59, 95% CI: -0.86 to -0.32).

Logistic regression for stress predictors was significant, χ²(7) = 17.968, *p* = 0.012, explaining 9.9% of the variance (Nagelkerke R² = 0.099) (Table 2). Significant predictors included “stress_concentration” (*p* = 0.004, OR = 0.547, 95% CI: 0.362–0.827), “stress_health” (*p* = 0.007, OR = 1.607, 95% CI: 1.141–2.262), and “highstress_selfmedication” (*p* = 0.030, OR = 1.392, 95% CI: 1.032–1.878). The classification accuracy was 68.9% (94.3% for self-medication and 21.4% for non-self-medication). High multicollinearity among the predictors was observed, which may have affected the model reliability.

Stress scores were further examined in relation to self-medication status (Fig. 3). Self-medicated students (*n* = 157) exhibited higher stress scores than non-self-medicated students (*n* = 84), consistent with the significant stress predictors identified in the regression model.

### Overall model and predictive performance

**Fig. 2:**
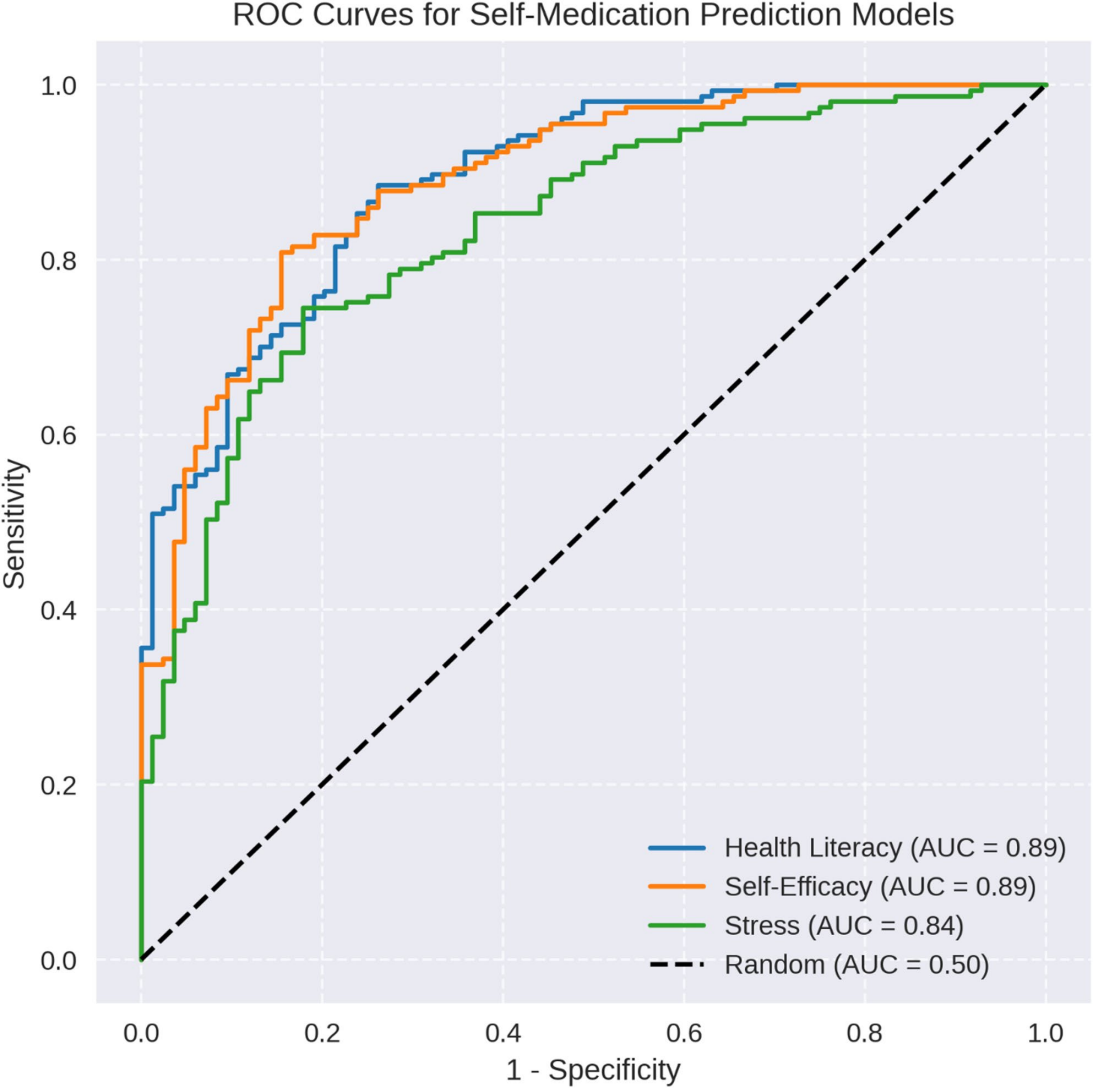
Receiver Operating Characteristic (ROC) curves for the logistic regression models predicting self-medication behaviour based on health literacy, self-efficacy, and stress among 241 medical students. The curves plot sensitivity against 1-specificity, with Area Under the Curve (AUC) values indicating model performance. The health literacy (AUC = 0.62) and self-efficacy (AUC = 0.66) models demonstrated moderate predictive ability, whereas the stress model (AUC = 0.58) performed less effectively. These curves illustrate the varying discriminatory powers of the models in identifying self-medication practices

The combined logistic regression model with 16 predictors was significant, χ²(16) = 34.740, *p* = 0.004, explaining 18.5% of the variance (Nagelkerke R² = 0.185). However, the classification accuracy was low (47.9–49.2%), and multicollinearity limited robustness. The predictive performances of the individual models are shown in Fig. 2.

## Discussion

**Fig. 3:**
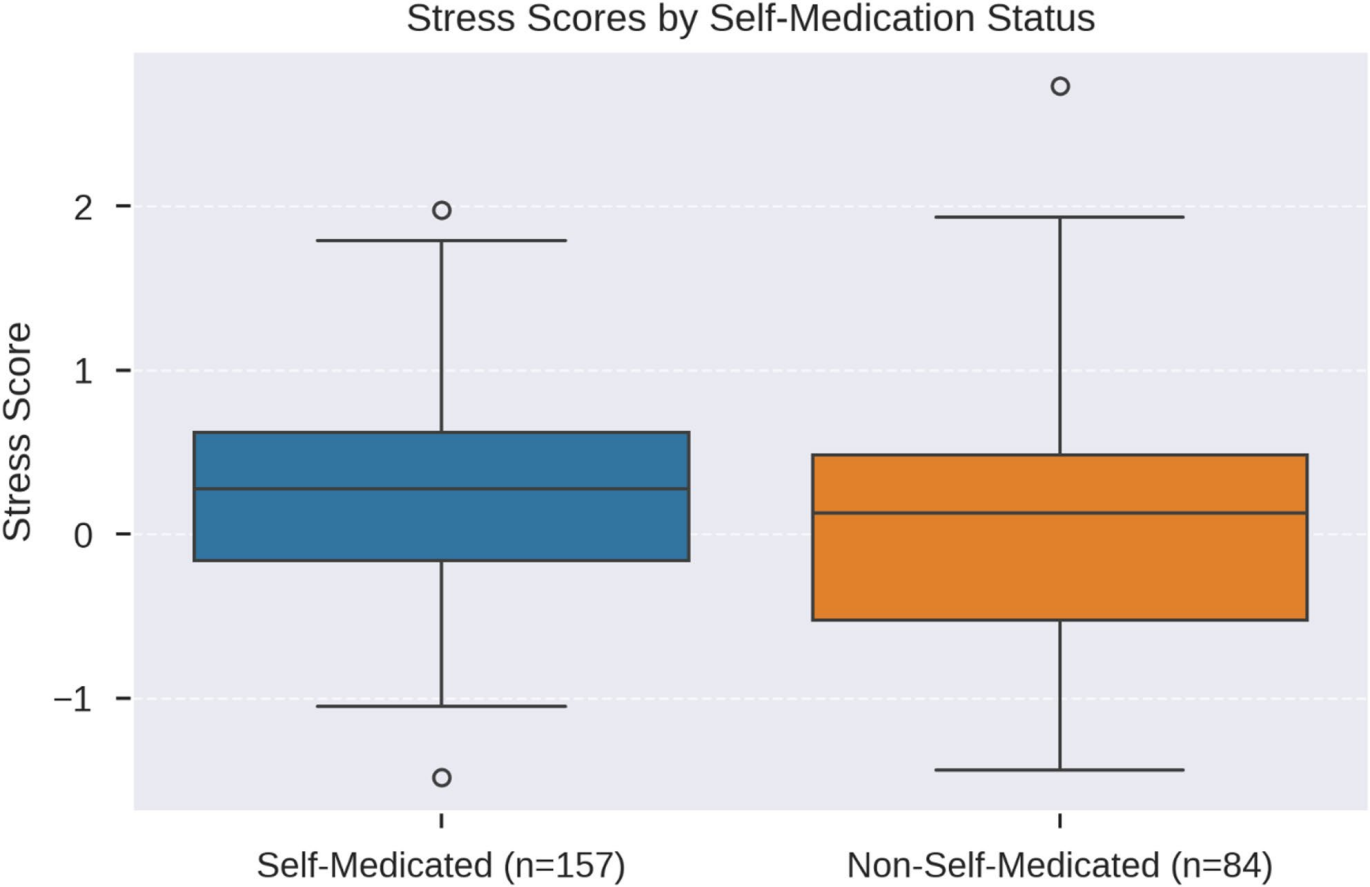
Box plot comparing stress scores between self-medicated (*n* = 157) and non-self-medicated (*n* = 84) students at Nowshehra Medical College. The plot displays the distribution of stress scores, including medians, interquartile ranges, and outliers. Self-medicating students showed higher stress scores, aligning with the significant stress predictors in the logistic regression model. This visualisation highlights the association between elevated stress and self-medication behaviours in this population

This cross-sectional study explored the association between stress, self-efficacy, health literacy, and self-medication among 241 medical students from Nowshehra Medical College, Pakistan. The findings provide valid data on what affects self-medication, an issue widely reported and connected to considerable public health implications [1]. The results indicated no differences between men and women in health literacy and self-efficacy, a substantial difference between men and women for stress in all models, and unsupervised medication use as the most important factor across all models. These results, displayed in Figs. 1, 2 and 3, reflect previous work and reveal contextually specific patterns among Pakistani medical students.

These findings should be interpreted within the framework of the Health Belief Model (HBM) and Bandura’s Self-Efficacy Theory, which guided this study. According to the HBM, perceived stress may trigger perceived behaviour by increasing the perceived need for immediate symptom relief, which ultimately contributes to self-medication behaviour. The idea is supported by an observed association between stress-related variables and self-medication in the present study. In contrast, Bandura’s Self-Efficacy Theory suggests that individuals with high confidence in managing health-related issues are more likely autonomous in making medication related decisions. This is consistent with our finding that self-efficacy-related variables such as reliance on past experience were significantly associated with self-medication behaviour.

The lack of gender differences in health literacy (*p* = 0.630, Cohen’s d = 0.07) suggests that male and female medical students possess comparable abilities to understand and apply health information [15]. This may reflect their shared academic training, which likely fosters uniform health literacy [8]. This underscores the ease of accessing over-the-counter drugs in Pakistan, which is a standard driver of self-medication [4, 23]. Studies in similar settings, such as India and Saudi Arabia, have reported comparable patterns, with 60–70% of medical students engaging in self-medication due to perceived knowledge and drug availability [7, 16]. Logistic regression analysis (Nagelkerke R² = 0.191) showed that health literacy (read_instruction) was not a significant predictor of self-medication behaviour (*p* = 0.772). Furthermore, the odds ratio (OR = 1.045; 95% CI: 0.775–1.409) did not indicate any meaningful association between health literacy and self-medication [5]. Similarly, self-efficacy showed no gender difference (*p* = 0.156, Cohen’s d = − 0.19), indicating that male and female students share similar confidence in managing health tasks [24]. The logistic regression model (Nagelkerke R² = 0.262) revealed that “rely_experiences” (OR = 1.823, *p* = 0.003) while “selfmedication_safe” was protective (OR = 0.679, *p* = 0.027). These results, depicted in Table 1, align with Bandura’s self-efficacy theory, which suggests that high confidence may lead to proactive but not always safe health behaviors [13, 25]. A study in Ethiopia found that students with high self-efficacy were 1.5 times more likely to self-medicate, often relying on their past experiences [14]. In Pakistan, the cultural acceptance of self-treatment and limited healthcare access may amplify this tendency [23].

The significant gender difference in stress levels (*p* < 0.001, Cohen’s d = -0.59), with females reporting higher stress (M = 0.39 vs. M = -0.02 for males), is consistent with the global literature on medical student well-being [6]. Figure 1’s bar chart vividly illustrates this disparity, which may stem from academic pressure, societal expectations, or gender-specific stressors [6]. The logistic regression model (Nagelkerke R² = 0.099) identified “stress_health” (OR = 1.607, *p* = 0.007) and “highstress_selfmedication” (OR = 1.392, *p* = 0.030) as significant predictors, while “stress_concentration” was a protective factor (OR = 0.547, *p* = 0.004). The box plot (Fig. 3) further shows higher stress scores among self-medicating students, suggesting that stress may drive self-medication as a coping mechanism [26]. Similar findings were reported in India, where 62% of students used non-prescribed analgesics for stress-related symptoms [7]. However, the high multicollinearity noted in the stress model warrants caution in interpreting these predictors [21].

The overall model (Nagelkerke R² = 0.185) explained a modest portion of the variance in self-medication with low classification accuracy (47.9–49.2%). The ROC curves in Fig. 2 (AUC: 0.58–0.66) indicate moderate predictive ability, with self-efficacy performing the best. This limited accuracy may reflect unmeasured factors, such as socioeconomic status or peer influence, which have been linked to self-medication in other studies [27]. The multicollinearity issue, particularly in the stress model, suggests overlapping predictor effects that can potentially inflate standard errors [28]. Future studies could employ variable reduction techniques, such as principal component analysis, to address this issue [9].

### Implications

These findings have significant implications for medical education and public health in Pakistan. The strong association between unsupervised medication use and self-medication calls for stricter regulations on over-the-counter drug sales. Educational interventions should enhance health literacy to promote safe practices, addressing the paradox of high literacy coexisting with risky behaviours. Stress management programs, particularly those addressing female students, could reduce reliance on self-medication for symptom relief. Additionally, fostering responsible self-efficacy through structured training may mitigate overconfidence in medication decision-making.

### Limitations

This study had several limitations. The non-probability convenience sampling limits the generalisability of the findings to other populations. Self-reported data may introduce recall or social desirability biases. The cross-sectional design precludes causal inferences, and unmeasured confounders (e.g. academic workload) may influence the results. High multicollinearity in the stress model suggests caution when interpreting the predictor effects. Finally, reliance on a self-modified questionnaire, despite pilot testing, may affect measurement validity.

### Future directions

Future research should employ longitudinal designs to establish the causality between stress, self-efficacy, health literacy, and self-medication. Validated instruments can enhance measurement reliability. Exploring additional predictors, such as cultural norms and access to healthcare, may improve model accuracy. Interventions targeting stress reduction and safe medication practices should be evaluated in randomised controlled trials. Comparative studies across medical and non-medical students could clarify whether medical training influences self-medication.

## Conclusion

This study highlights the intricate relationship between stress, self-efficacy, and health literacy in the context of self-medication among medical students at Nowshera Medical College. These findings underscore the necessity for targeted interventions to promote safe health practices and address gender-specific stressors, thereby contributing to enhanced health outcomes in this critical population.

## Supplementary Information

Supplementary Material 1.

## Data Availability

The datasets generated and analyzed during the current study are not publicly available due to participant confidentiality but are available from the corresponding author upon reasonable request.

## Abbreviations

AUC: Area Under the Curve
CI: Confidence Interval
ERB: Ethical Review Board
HL: Health Literacy
NMC: Nowshehra Medical College
OR: Odds Ratio
OTC: Over-the-counter
ROC: Receiver Operating Characteristic
SD: Standard Deviation
SPSS: Statistical Package for the Social Sciences
WHO: World Health Organization
