# Supplementary material for: Self-medication behavior among medical students: the roles of stress, self-efficacy, and health literacy
Source: BMC Med Educ. 2026 Apr 23;26:919. doi: 10.1186/s12909-026-09240-5 (PMC13238113; doi:10.1186/s12909-026-09240-5)
Supplement: Supplementary file 1 — Supplementary Material 1. [file 12909_2026_9240_MOESM1_ESM.docx]

Supplementary file 1 : Questionnaire

# Demographics:

Age: Gender: Year of study:

Have you ever self–medicated? Yes/No

# Q1. I frequently experience high levels of stress.

a)strongly disagree b)disagree c)neutral d)agree e)strongly agree

# Q2. Stress affects my ability to make health related decisions.

a)strongly disagree b)disagree c)neutral d)agree e)strongly agree

# Q3. I understand how different medications work.

a)strongly disagree b)disagree c)neutral d)agree e)strongly agree

# Q4. I often take medication without consulting a healthcare professional.

a)strongly disagree b)disagree c)neutral d)agree e)strongly agree

# Q5. I rely on past experiences when deciding on self-medication.

a)strongly disagree b)disagree c)neutral d)agree e)strongly agree

# Q6. I seek information from the internet before taking medication.

a)strongly disagree b)disagree c)neutral d)agree e)strongly agree

# Q7. I believe self-medication is a safe practice if done correctly.

a)strongly disagree b)disagree c)neutral d)agree e)strongly agree

# Q8. I understand medication labels and instructions clearly.

a)strongly disagree b)disagree c)neutral d)agree e)strongly agree

# Q9. I feel confident in identifying symptoms of common illnesses.

a)strongly disagree b)disagree c)neutral d)agree e)strongly agree

# Q10. I believe self-medication can sometimes be more convenient than seeing a doctor.

a)strongly disagree b)disagree c)neutral d)agree e)strongly agree

# Q11. I read about drug interactions before taking multiple medications.

a)strongly disagree b)disagree c)neutral d)agree e)strongly agree

# Q12. I am aware of the risks of self-medication.

a)strongly disagree b)disagree c)neutral d)agree e)strongly agree

# Q13. I keep track of the medications I take to avoid misuse.

a)strongly disagree b)disagree c)neutral d)agree e)strongly agree

# Q14. I find it easy to understand health-related information from doctors or pharmacists.

a)strongly disagree b)disagree c)neutral d)agree e)strongly agree

# Q15. I believe healthcare professionals should always be consulted before taking any medication.

a)strongly disagree b)disagree c)neutral d)agree e)strongly agree

# Q16. I have faced negative effects due to self-medication in the past.

a)strongly disagree b)disagree c)neutral d)agree e)strongly agree

# Q17. I would prefer professional healthcare guidance over self-medication in most cases.

a)strongly disagree b)disagree c)neutral d)agree e)strongly agree

# Q18. I often feel overwhelmed by my daily responsibilities.

a)strongly disagree b)disagree c)neutral d)agree e)strongly agree

# Q19. Stress negatively impacts my sleep quality.

a)strongly disagree b)disagree c)neutral d)agree e)strongly agree

# Q20. I find it difficult to relax even when I have free time.

a)strongly disagree b)disagree c)neutral d)agree e)strongly agree

# Q21. High stress levels make me more likely to take self-medication for minor issues.

a)strongly disagree b)disagree c)neutral d)agree e)strongly agree

# Q22. I experience frequent mood changes due to stress.

a)strongly disagree b)disagree c)neutral d)agree e)strongly agree

# Q23. I feel that stress affects my ability to concentrate on health-related decisions.

a)strongly disagree b)disagree c)neutral d)agree e)strongly agree

# Q24. I have tried different coping strategies to reduce my stress.

a)strongly disagree b)disagree c)neutral d)agree e)strongly agree

# Q25. My stress levels have influenced my overall health in the past.

a)strongly disagree b)disagree c)neutral d)agree e)strongly agree
